# Quantifying navigational information: The catchment volumes of panoramic snapshots in outdoor scenes

**DOI:** 10.1371/journal.pone.0187226

**Published:** 2017-10-31

**Authors:** Trevor Murray, Jochen Zeil

**Affiliations:** Research School of Biology, Australian National University, Canberra, Australia; University of Sussex, UNITED KINGDOM

## Abstract

Panoramic views of natural environments provide visually navigating animals with two kinds of information: they define locations because image differences increase smoothly with distance from a reference location and they provide compass information, because image differences increase smoothly with rotation away from a reference orientation. The range over which a given reference image can provide navigational guidance (its ‘catchment area’) has to date been quantified from the perspective of walking animals by determining how image differences develop across the ground plane of natural habitats. However, to understand the information available to flying animals there is a need to characterize the ‘catchment volumes’ within which panoramic snapshots can provide navigational guidance. We used recently developed camera-based methods for constructing 3D models of natural environments and rendered panoramic views at defined locations within these models with the aim of mapping navigational information in three dimensions. We find that in relatively open woodland habitats, catchment volumes are surprisingly large extending for metres depending on the sensitivity of the viewer to image differences. The size and the shape of catchment volumes depend on the distance of visual features in the environment. Catchment volumes are smaller for reference images close to the ground and become larger for reference images at some distance from the ground and in more open environments. Interestingly, catchment volumes become smaller when only above horizon views are used and also when views include a 1 km distant panorama. We discuss the current limitations of mapping navigational information in natural environments and the relevance of our findings for our understanding of visual navigation in animals and autonomous robots.

## Introduction

In natural environments, the local panorama defines places and in addition provides compass information ([1–3], reviewed in [4]). Most generally, this is due to the fact that panoramic image differences caused by translation increase smoothly with distance from the location of a reference image (translational image difference function–transIDF) and those caused by rotations around the yaw-axis increase smoothly with rotation relative to the reference orientation (azimuthal rotational image difference function–rotIDF). The shape of the resulting image difference functions in the case of the transIDF depends on the distance distribution of visible features in the scene. The shape of the rotIDF depends on the spatial frequency content of the scene [2], on the presence of vertical edges [5] and on the contribution of distant visual features to the panorama. The range over which a given reference image can provide navigational guidance to location depends on the distance distribution of objects in a given landscape, with the transIDF gradient being steeper at a location with nearby landmarks that cause the panoramic scene to change more rapidly with translation, compared to a panoramic view in an open environment with distant landmarks [1, 2]. In cluttered environments, the ‘catchment’ of a reference view, defined as the distance from the reference location at which a gradient descent in image differences would successfully converge onto the minimum of the transIDF, is also smaller, compared to open environments [2]. The range over which a given reference image can provide compass information is defined by those locations where the rotIDF has a detectable minimum when the reference image is rotated against the current view. This range depends on the fraction of the image occupied by distant landscape features, such as mountains and thus is smaller in cluttered environments where nearby objects obstruct the views to distant features, compared to open environments where such features can be seen from any vantage point. Image differences due to rotation are generally larger than those due to translation because all image regions contribute equally to the rotIDF (rotational optic flow) while image differences due to translation are dependent on the distance of visual features (translational optic flow). Therefore, the rotIDF minimum needs to be found first so that the transIDF can be minimized through translational movements only (see [1, 6]). The degree to which navigating animals can exploit this information also depends on their sensitivity to global image differences and the resolving power of their visual system (e.g. [3, 7]).

The usefulness of both transIDF and rotIDF for navigation depends on the fact that natural scenes tend to be unique, with little danger of similar views existing in different places. In a scene that only contains four identical and equidistant landmarks, for instance, the rotIDF would have four indistinguishable minima in four compass directions and thus would present a serious problem with aliasing. However, such repetitive or symmetrical scenes may be common in human built environments, such as corridors in office buildings, but are extremely rare in nature.

Habitat structure thus determines the navigational information available to a visually navigating animal, both in terms of places and routes [1, 3, 4, 8–11]. The navigational information content of natural habitats can be mapped and quantified, but so far with two exceptions [1, 12] this has only been done in the horizontal plane in natural (e.g. [1–3, 12–14]) in experimental [15–17] and in simulated environments [7, 10, 11, 18, 19]. However, the navigational guidance by panoramic views also extends in the vertical direction (see [1, 13]), so that a full description of the information they contain must be in terms of catchment volumes.

Here, we make use of recent developments in camera-based 3D modelling of natural environments to extend previous analyses of the navigational information content of habitats (see [3]) to also cover the third dimension. We describe the catchment volumes using systematic grids of panoramic views rendered in 3D models of three environments, which differ in the three-dimensional layout of visually conspicuous vegetation.

## Materials and methods

### 3D modelling and image rendering

**Fig 1:**
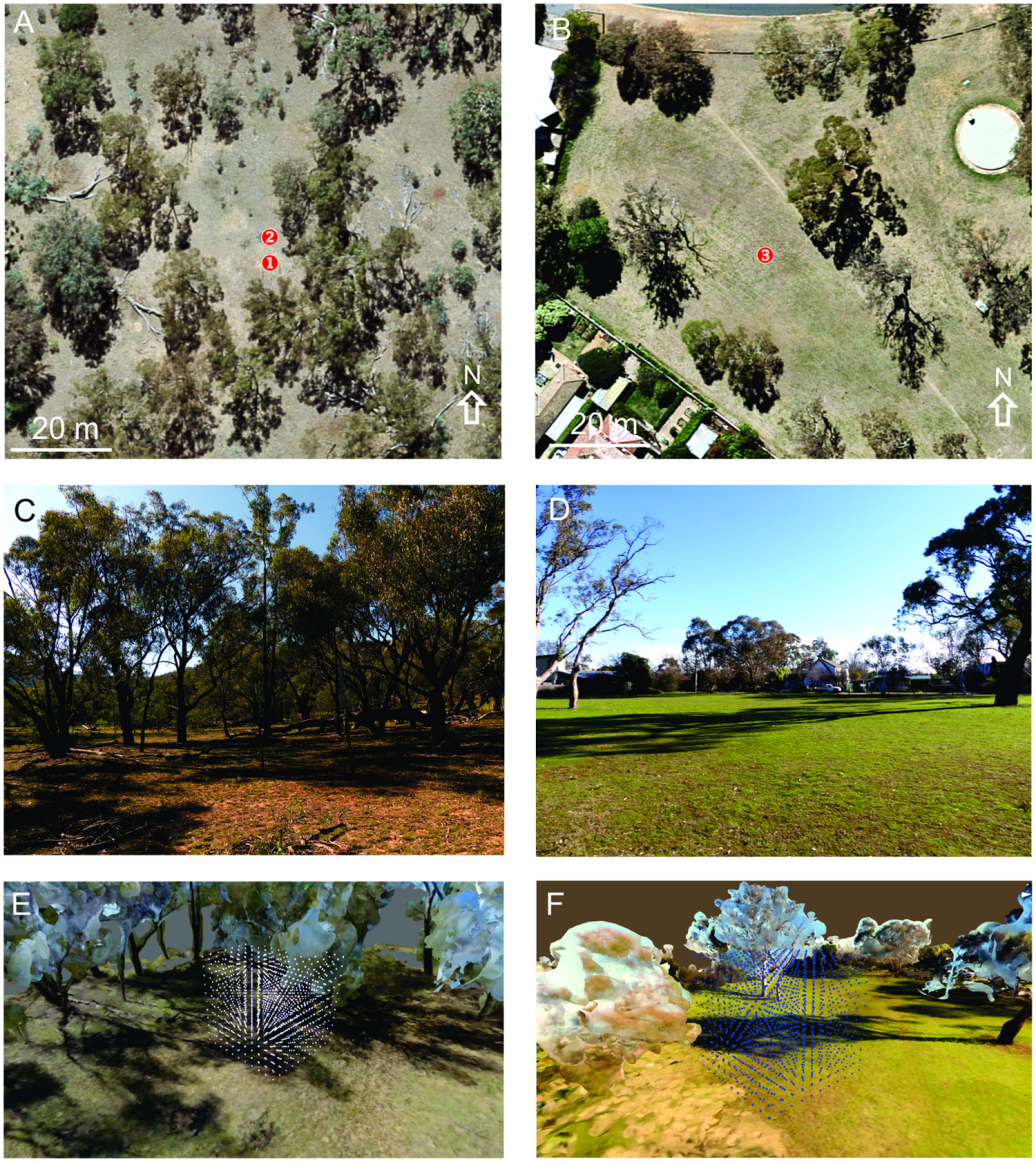
The three experimental habitats in aerial photographs (top row), in photographs on the ground (middle row) and viewed as 3D models (bottom row). **(A)** Sites 1 and 2 within Mount Majura Nature Reserve with photograph looking east in C and north-west in E. **(B)** Site 3 in Hackett Park, with photograph looking north in D and north-west in F. Note 5 m^3^ panoramic image sampling grids indicated in E and 10 m^3^ in F. Aerial photographs from ACT Mapi, 2016 series available under Creative Commons Attribution 4.0 International Licence.

We took an overlapping image series of three habitats using a Panasonic DMC-FZ200 digital camera at 4000x3000 pixel resolution. At 10:30 on 07/03/2016 we recorded the environment (Site 1 & 2, Fig 1A and 1C) around the nesting aggregation of ground-nesting wasps (genus *Cerceris*, the Mount Majura Nature Reserve, Australian Capital Territory, Canberra, Australia (35°14'37.01"S; 149°10'10.72"E); see Stürzl et al. 2016) and at 9:15 on 03/08/2016 we recorded an urban park (Site 3, Fig 1B and 1D) where we analyse ant navigation (35°15'05.59"S, 149°09'33.18"E), Canberra, Australia (see [3, 14, 20]). To generate robust distance information with camera-based 3D modelling it is important to generate sharp images (short shutter speeds) from a sequence of close vantage points to ensure enough overlap and to translate the camera over sufficiently large distances in different directions across the whole image series (see [3]). Our image sampling regime aimed to capture all terrain features visible from the area, focusing mainly on the ground and the trees surrounding the nesting site. Images were used as input to a 3D reconstruction program (Pix4DMapper, Pix4D SA, Lausanne, Switzerland) to produce a point cloud and a 3D model with a 1 000 000 polygon limit and 4168x4168 texture size (see Fig 1E and 1F). The 3D model was exported to Unity3D (Unity Game Engine-Official Site (http://unity3d.com), v5.3.4f1 released December 8, 2015). All lighting options were deactivated except for a white global ambient light to ensure the 3D model best matched the natural conditions captured in the images. Graphics settings were set to fastest, giving a low texture resolution, which is sufficient for our purpose to quantify the navigational information available to low-resolution insect eyes.

Within these models we rendered panoramic views by simulating six cameras with 95°x95° fields of view and 128x128 pixel resolution facing north, east, south, west, up, and down. We exported camera images as a png files and spherically projected the cube-map they form onto a 2D image using previously developed methods by Stürzl et.al. [3] in OpenCV [21]. The resulting panoramic views cover 384 pixels in horizontal direction and 192 pixels in vertical direction (thus representing the view sphere with 0.9° resolution). The red, green, and blue colour channels of the rendered panoramas were flattened to 8 Bit greyscale using OpenCV’s [21] cvtColor with the CV_BGR2GRAY argument. We rendered such panoramic views from within the 3D models at regularly spaced locations in a three-dimensional grid from ground-level to a height of up to 10 m. The panoramic views are aligned with the sampling grid such that the x-axis points east, the y-axis north and the z-axis vertically up.

### Measuring navigational information

We generated three sets of panoramas, one at 10 cm intervals for a 5 m x 5 m x 5 m cube centred 2.5 m above a ground-nesting wasp nesting aggregation (Site 1, Fig 1A, 1C and 1E; 51 x 51 x 51 panoramas), the second larger cube (10 m x 10 m x 10 m) with 20 cm intervals in a more open area 5 m north of the nesting aggregation (Site 2, Fig 1A), and the same in an open woodland park (Site 3, Fig 1C, 1D and 1F). We then selected from this set of panoramas reference images in the centre of the grid at different heights above ground and calculated the r.m.s. pixel difference between these reference images and all images in the cube. Reference images represent a remembered snapshot that an insect may take at a given location that it later compares to its current view.

**Fig 2:**
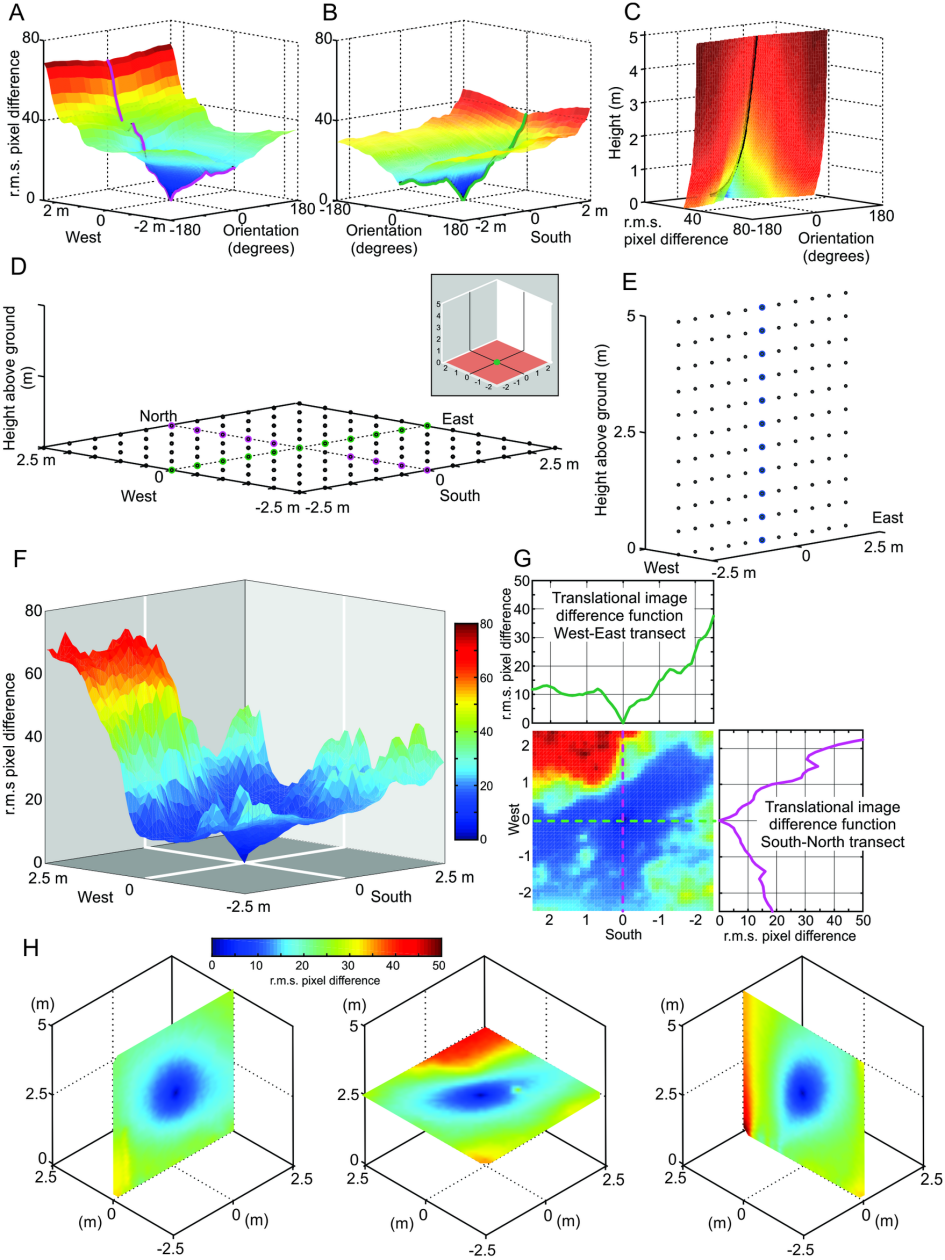
The relationship between rotational and translational image difference functions. **(A)** The rotational image difference functions (r.m.s. pixel differences) of panoramic images taken every 10 cm along a north-south transect marked mauve in (D) compared to a reference image in the centre. The contour created by their minima mark out the translational image difference function along that transect (highlighted in mauve. **(B)** Same as (A), but along an east-west transect marked green in (D). **(C)** Rotational image difference functions along a vertical transect marked in blue as indicated in (E). **(D)** Schematic map of panoramic image locations in a horizontal grid in the ground plane. Coloured transects correspond to (A) and (B) and insect shows reference image location. **(E)** Schematic map of a vertical grid with highlighted transect corresponding to (C). **(F)** The translational image difference function (transIDF) for the ground-plane grid shown in (D) with r.m.s. pixel differences on the vertical axis and the horizontal location of the panoramic grid image on the x- and y-axis. **(G)** Top-down plane view of the transIDF shown in (F) with colour-coded transects on the top and to the right corresponding to those shown in (A) and (B). Colour bar scale in (F) for all panels. **(H)** Transects along each dimension through the Image Difference Function (transIDF) for a reference image at 2.5 m above ground at site 1. Note that the transIDF is a three-dimensional structure with values increasing gradually away from the reference image location in all directions.

To determine the navigational information available in these habitats we compared reference panoramas taken, for instance, at the ground to the views rendered at regular grid locations in a cube above or around the reference location. We compared pairs of panoramas with the same orientation by calculating the root mean squared pixel difference across the whole set of images which results in a three-dimensional image difference function that is due to translation only covering the grid volume (transIDF, see [1, 2]). It should be noted that all panoramas have the same orientation as the reference image and that our reconstructions have consistent illumination, lack environmental motion, and do not contain repetitive features, which would lead to significant false minima. We show the surfaces of rotIDFs for two transects across a ground plane horizontal grid slice in Fig 2A, 2B and 2D and for a vertical transect in Fig 2C and 2E. The valley contours connecting the minima of rotIDFs are marked by a mauve and a green line which constitute the transects through the transIDF for this grid of images (shown as 3D surface in Fig 2F and in a top-down view in Fig 2G). Note that the transIDF is a three-dimensional structure with values increasing smoothly from the location of the reference image (Fig 2H).

### Determining catchment volumes

We used a hill climbing algorithm to determine the volume within which a single reference image provides guidance to its location. This algorithm searched for the steepest descending gradient in the image difference function between the current and neighbouring grid-points before moving to that neighbouring grid-point and repeating the search. We determined the steepness of the gradient by comparing the r.m.s. pixel differences relative to the reference image between the current and neighbouring spatial grid points in the x-, y-, z-directions, including diagonals. Formally, if *V*_1_ is the r.m.s. pixel difference of the current grid-point, *V*_*n*_ is the r.m.s. pixel difference of a neighbouring grid-point, and *distance* is the spatial distance between the two grid-points, then:

$$descending\;gradient=\frac{V_{1}-V_{n}}{distance}>0$$

$$distance=\sqrt{{{(V}_{1x}-V_{nx})}^{2}+{{(V}_{1y}-V_{ny})}^{2}+{{(V}_{1z}-V_{nz})}^{2}}$$

We ran hill climbs starting from every grid-point in the volume. In addition to finding the steepest downwards gradient, we imposed a threshold value that the gradient had to exceed in order to progress. We selected this threshold as 10% of the maximum of the rotational image difference of the reference image rotated against itself, on a per meter basis. This provided a relative measure accounting for local image structure. The grid-points from which the hill climb reached the reference image location were marked as successes; those that left the volume, ended in false minima, or ended when they failed to exceed the threshold were marked as failures. The resulting point cloud shows all 3D locations (the catchment volume) from which a gradient descent in image differences would converge onto the location of the reference image, assuming the ability to detect image differences as small as the defined threshold. We used a hill climbing algorithm to determine the volumes shown in this manuscript for computational efficiency and because it allowed us to implement a threshold. In addition, we ran both hill climbing and gradient descent algorithms (without threshold) from random locations within our image grids to demonstrate that our procedure correctly identifies the presence of a continuous gradient and that hill climbing and gradient descent produce comparable results (see S1 Fig).

### Comparing models with and without distant background features

It is important to note that camera-based 3D models of natural environments have a finite extent that is limited by the area covered by the camera during image capture. In order to integrate views from inside the model with the distant panorama, we projected a panoramic view taken 1m above the ground plane of the recording area into the inside of a 1 km diameter sphere which encased the 3D model, centred at the recording location. The panorama was constructed from a series of images taken with a camera (Panasonic DMC-FZ200) rotating 360° around its vertical axis at three pitch angles pointing 45° downward, horizontal and pointing 45° upward. We stitched the images captured along these three slices into a single panoramic image using Hugin [22] to create the background panorama. We document the effect of this distant panorama on the navigational information provided by rendered panoramic images by comparing rendered views containing the distant panorama with those where the distant panorama was uniform blue.

## Results

### Visualizing catchment volumes

**Fig 3:**
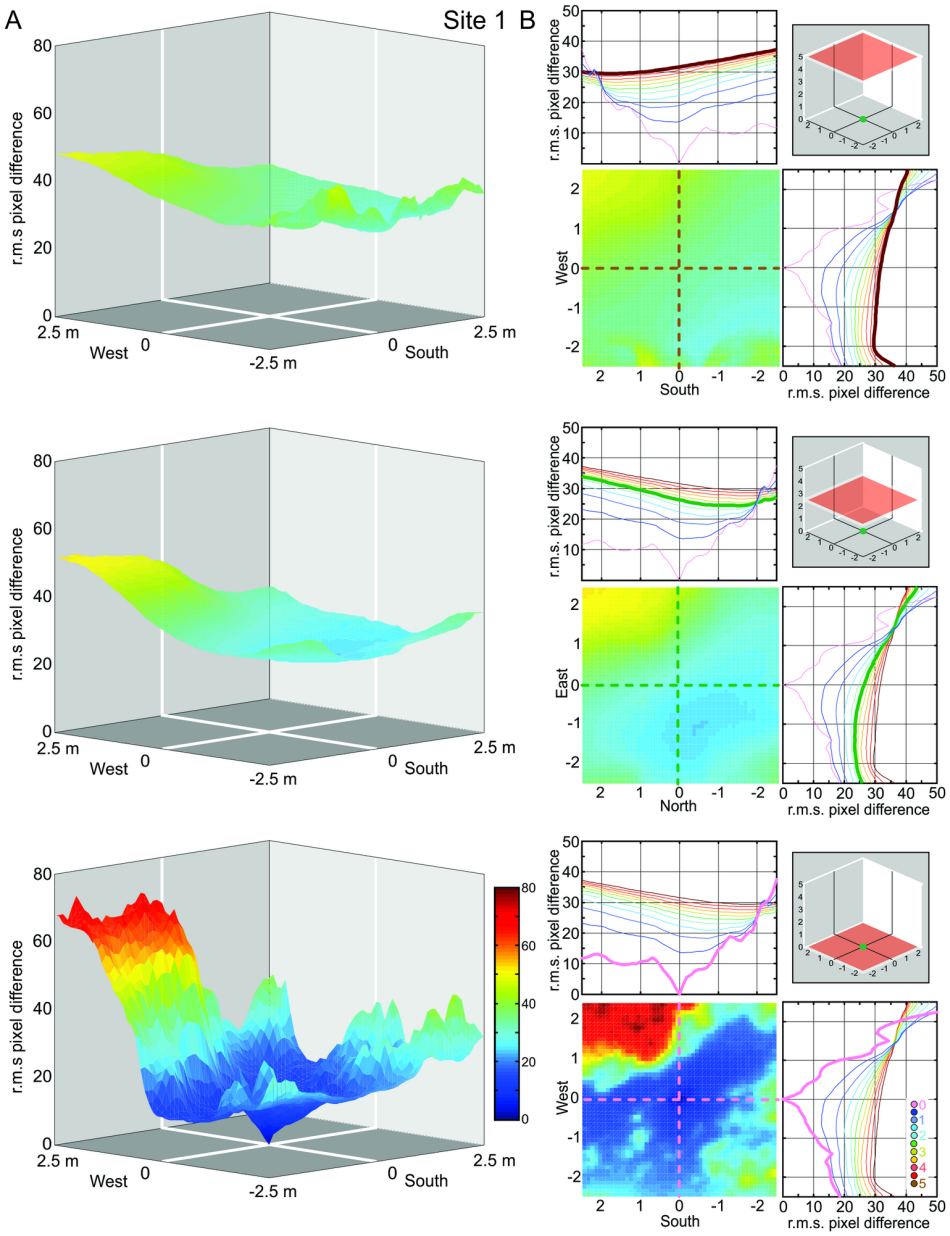
The image difference functions (transIDF) along horizontal planes at site 1: Reference image at ground-level. **(A)** Plots show the root mean squared (r.m.s.) pixel difference between the reference image on the ground and each panoramic image at height 5 m (top), 2.5 m (middle) and 0 m (bottom) in a 5m by 5m grid centred on the reference location, with r.m.s. pixel differences on the vertical axis and the horizontal location of the panoramic grid image on the x- and y-axis. Reference image location (green dot) and the plane of comparison images (red square) are indicated in the right-most inset in B. **(B)** Top-down plane views of the IDFs shown in (A), together with north-south and east-west transects (indicated by dashed lines in top-down plane views) through the IDFs for planes spaced at 50 cm height intervals which are colour coded as indicated by dot inset in bottom graph. Transects corresponding to the three IDF planes shown in full in (A) and (B) (as indicated by inset schematics in (B) are highlighted by thick lines (brown, green and pink from top to bottom in (B).

**Fig 4:**
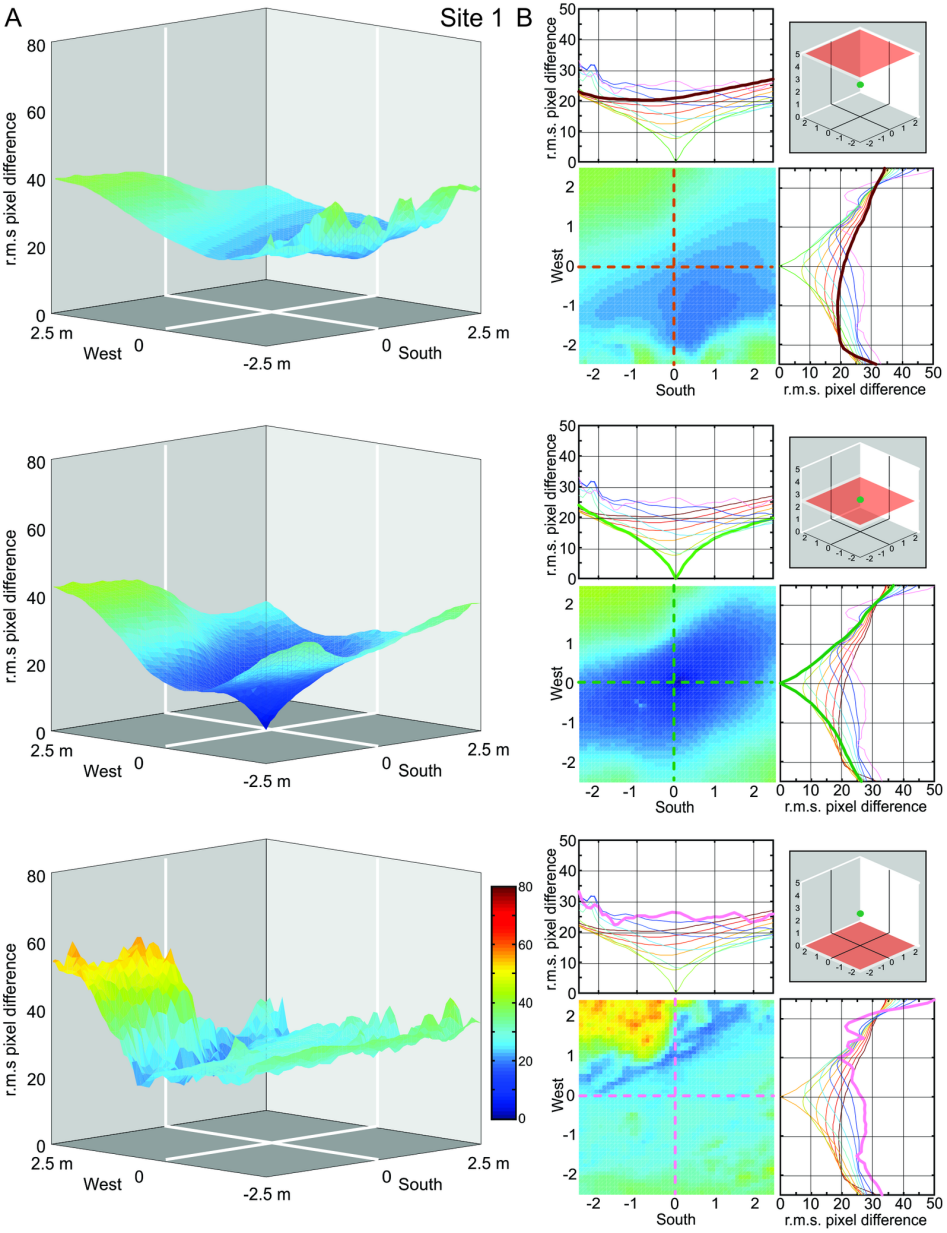
The image difference functions (transIDF) along horizontal planes at site 1: Reference image at 2.5 m above ground. Otherwise conventions as in Fig 3.

**Fig 5:**
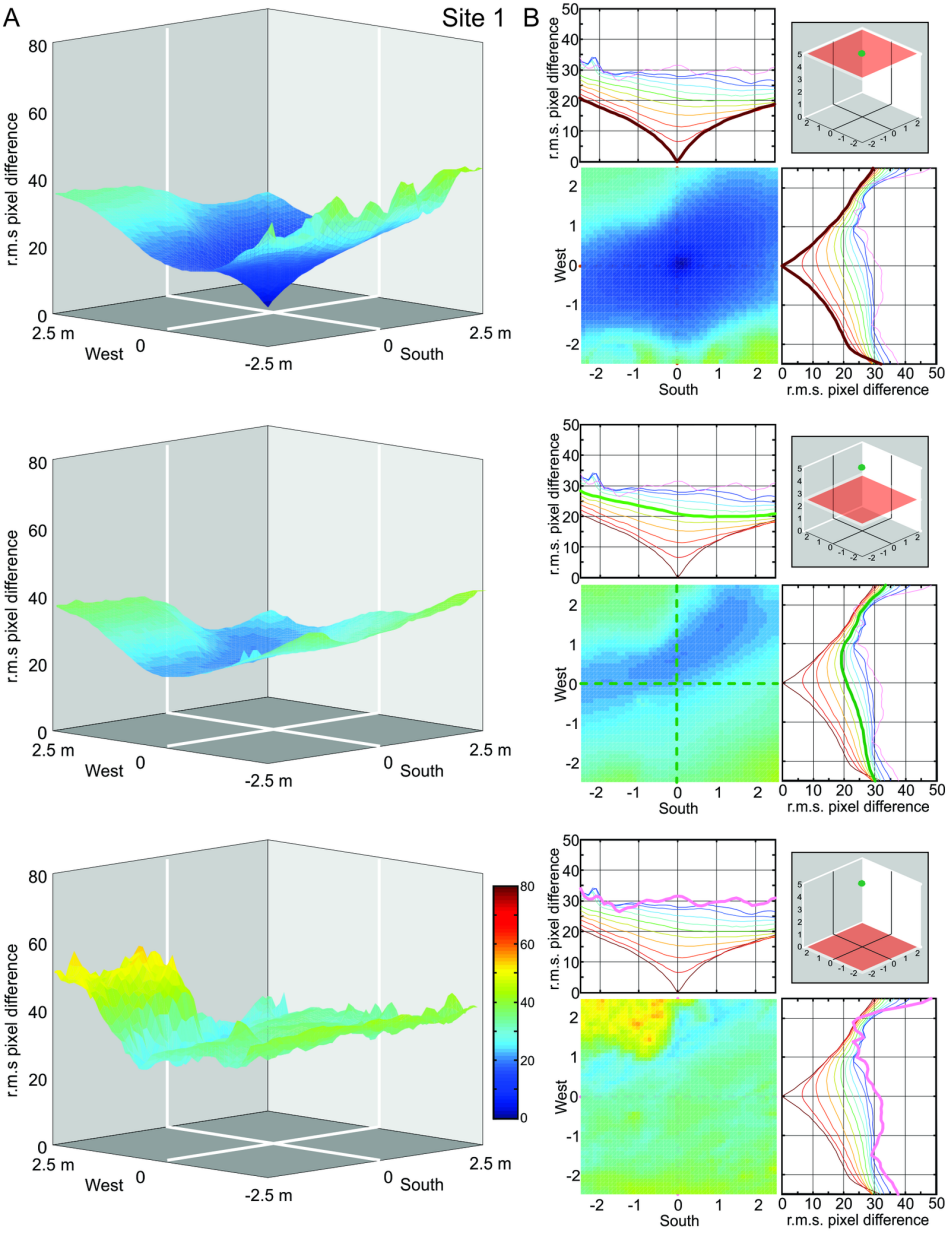
The image difference functions (transIDF) along horizontal planes at site 1: Reference image at 5 m above ground. Otherwise conventions as in Fig 3.

Our procedure to determine the navigational information provided by a panoramic snapshot (its catchment volume) results in a 3D matrix of 51^3^ image difference values (r.m.s. pixel differences) with 10 cm or 20 cm spacing. We first visualize the structure of these matrices at Site 1 by presenting the image difference function along three horizontal sections at 0 m, 2.5 m, and 5 m above ground for three different heights of the reference image (Figs 3, 4 and 5, respectively). We will later determine the volume of detectable gradients in image differences, but will concentrate first on analysing the distribution of image differences in space.

### The horizontal distribution of image differences at different heights above ground

**Fig 6:**
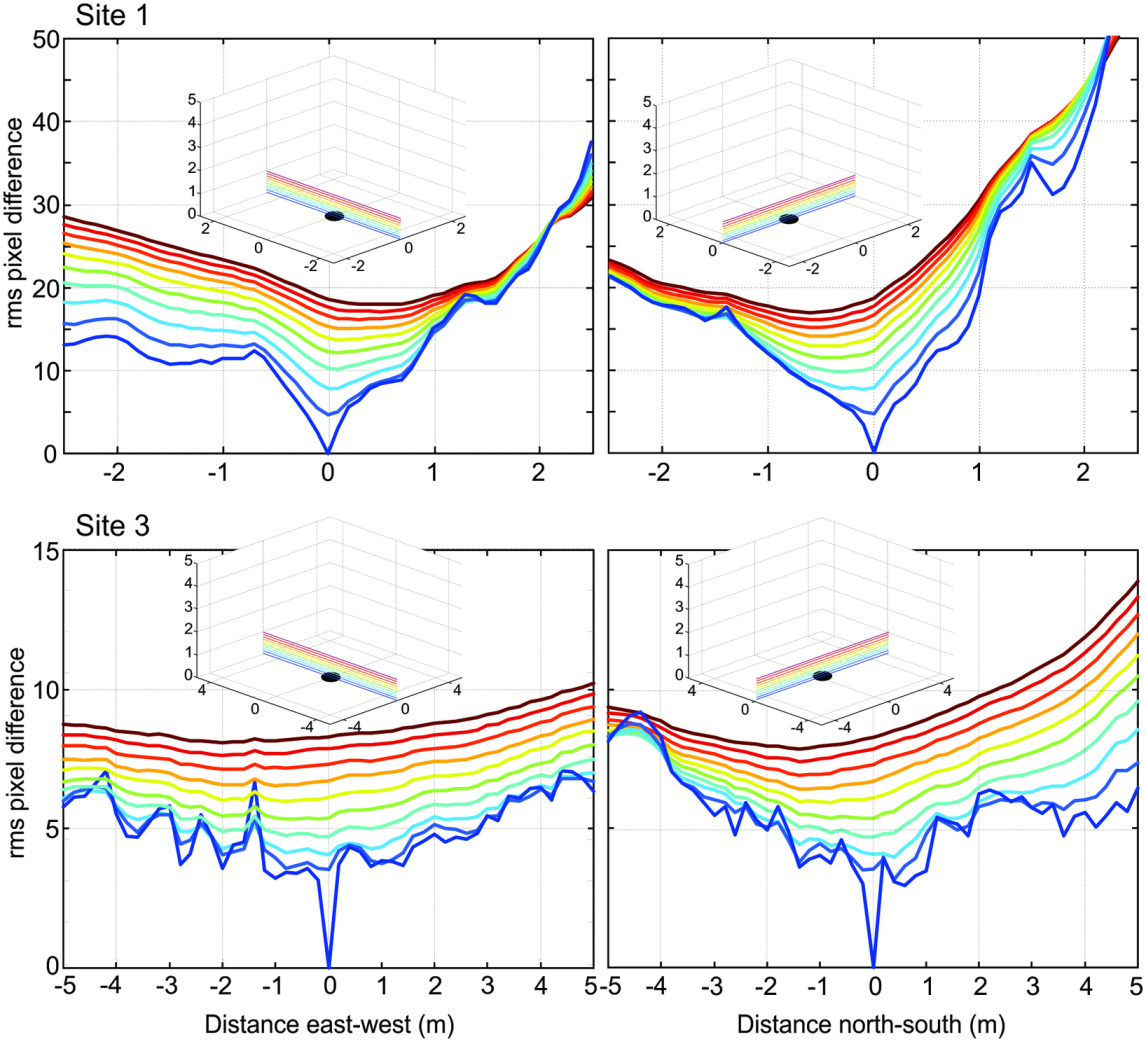
The change of image difference functions (transIDF) close to the ground at site 1 and site 3. For the reference image at ground level, east-west and north-south transects through the transIDF are shown at 10 cm height intervals for site 1 and 20 cm intervals for site 3 as indicated by insets.

We start by discussing the situation at our Site 1 (see Fig 1). For all three reference images (column A in Figs 3–5), the image difference surface on the ground plane (bottom row) is quite rugged, containing potentially numerous small ‘false’ local minima, with a ‘cliff’ on the left (north-western) side. Considering the fact that IDFs become smoother in horizontal planes above the ground (top and middle row in Figs 3–5), note that this ruggedness is due to the closeness of visual features on the ground (including shadows) which cause images to change rapidly with translation parallel to the ground (see analysis below which compared compares transects with and without ground features). The north-western cliff in the IDF is caused by image positions beyond the shadow cast by a nearby tree, which results in the ground being much brighter due to direct sunlight. Note also that despite the rugged IDF close to the ground for the ground-level reference image (bottom panel column A in Fig 3) there is still a local cusp-shaped IDF gradient in the vicinity of the reference location (shown in detail and enlarged in Fig 6).

The shape of the IDF due to translation (transIDF) thus depends on the three-dimensional layout of objects in the environment [2] and this can be demonstrated here by comparing the image difference between the ground-level reference image with those along transects in steps of 10 or 20 cm height above ground between 0 cm and 50 cm or 1 m (Fig 6). Overall, the IDF becomes smoother with distance from the ground as close features on the ground cease to contribute significantly to image differences. Note also that the cusp-shaped gradient around the reference image location becomes shallower as height above ground increases and that the horizontal location of the IDF minimum shifts away from the location of the reference image (Figs 3–6).

### The image difference functions in different environments

We repeated the analysis at two further sites, since it appears that the close proximity of Site 1 to nearby trees and their shadows have a large effect on the shape of the IDF. It also turned out that the 5 m cube was not sufficient to reach the plateau of the IDF. One of these sites is 5 m north of Site 1 and further from the edge of the clearing (Site 2, Fig 1A) and the other at an open woodland park with a few large scattered trees (Site 3, Fig 1B).

**Fig 7:**
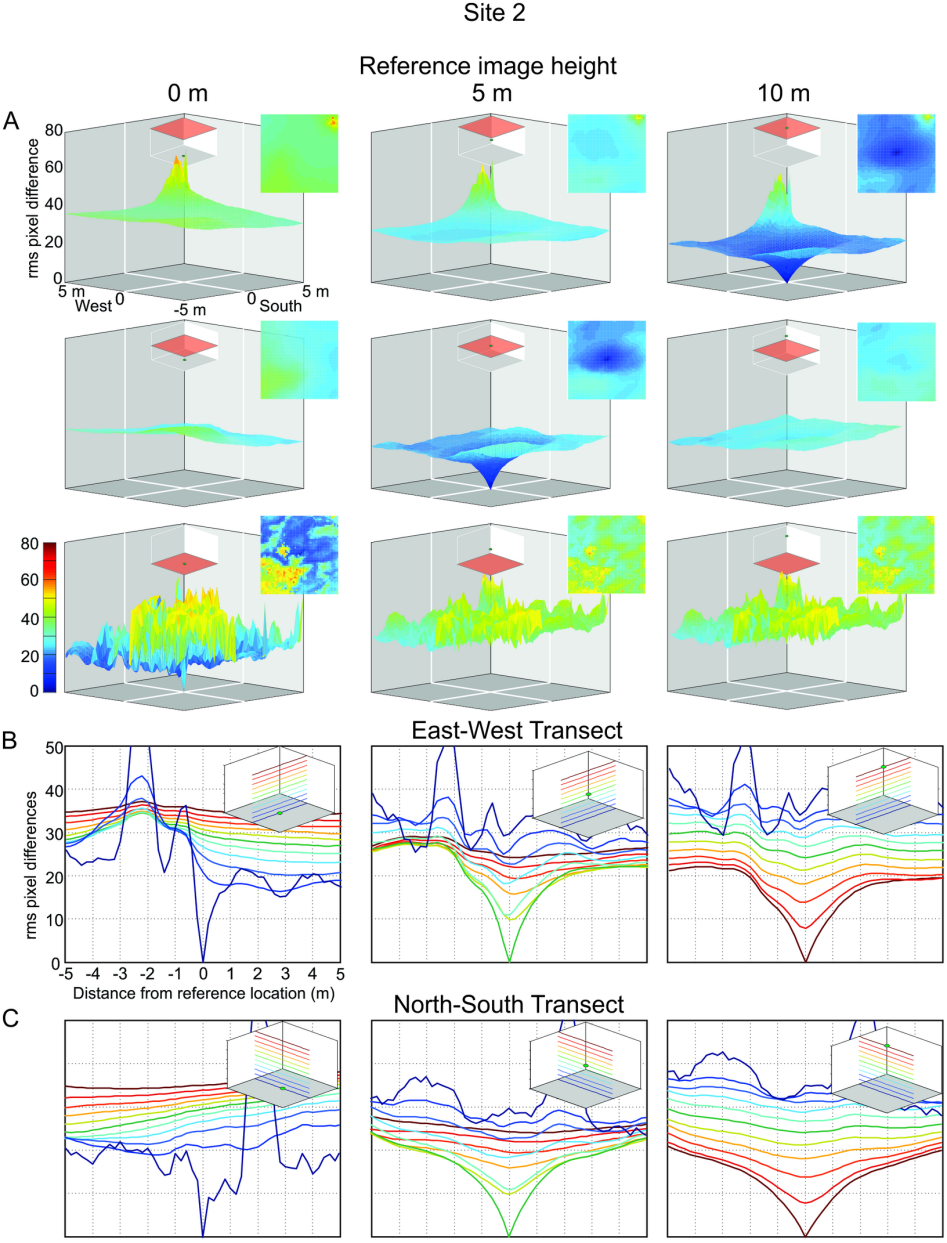
The image difference functions (transIDF) at site 2. **(A)** Panels show the transIDF for reference images at ground level (left column), at 5 m and at 10 m above ground (middle and right column) with comparison image transect locations as indicated by insets. Top-down plane views of the IDF are shown on the right of each panel. **(B)** Transects through the IDFs shown in (A) along the east-west direction colour-coded as indicated by insets. **(C)** Same for north-south transects.

At these sites we were able to generate a 10 m^3^ grid of panoramic images in an attempt to capture the full extent of catchments. At Site 2 we find that the transIDF does plateau within a 5 m radius of the reference location in the East-West direction (Fig 7B), but not in the North-South direction (Fig 7C), again emphasizing the degree to which the layout of objects in the environment determines the shape of image difference functions. Note also how image differences of panoramic snapshots taken near the ground do not increase smoothly with distance from the reference location, except in the close vicinity of the reference location. In this particular case this is due to ground vegetation and strong shadows (dark blue traces in bottom panels Fig 7).

**Fig 8:**
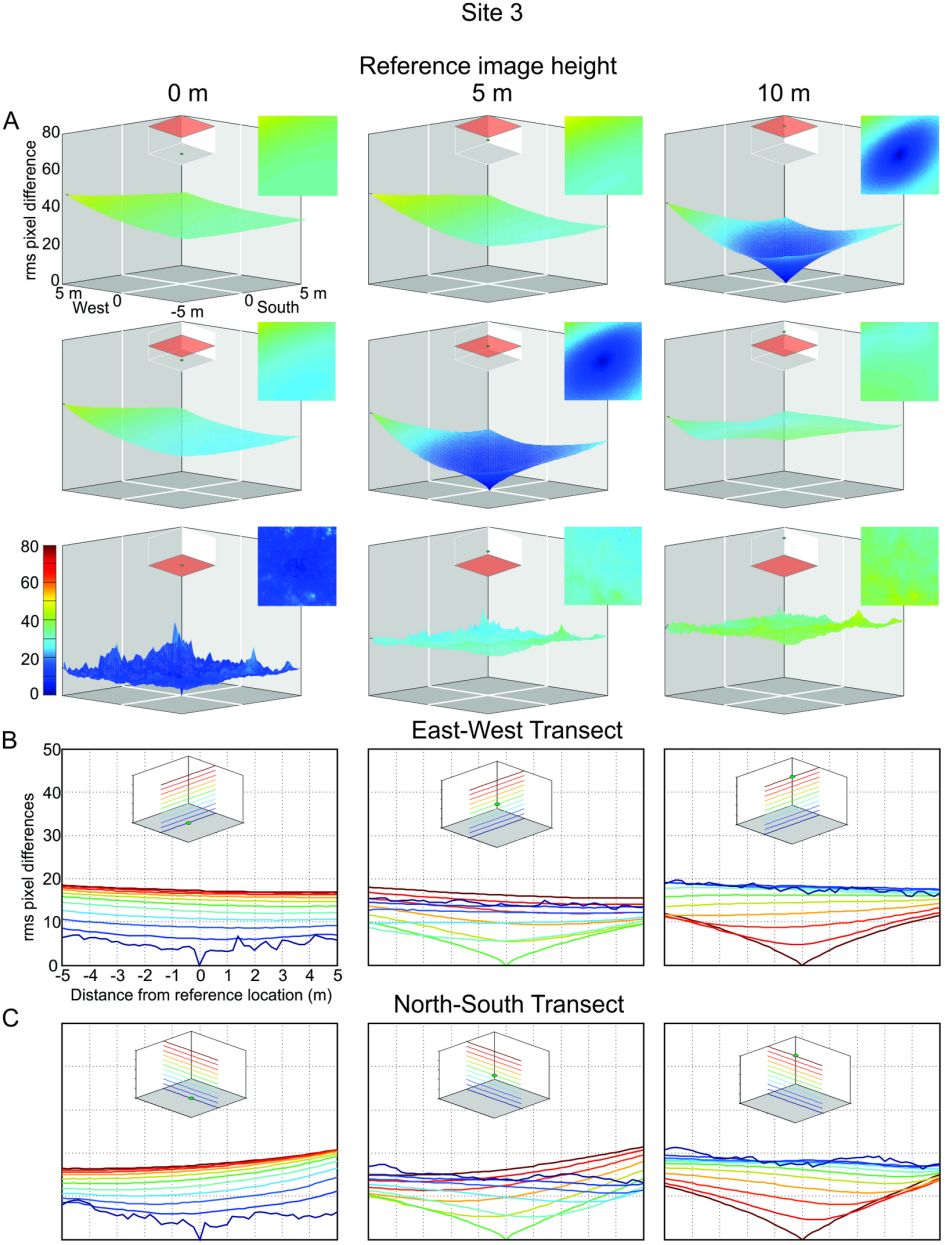
The image difference functions (transIDF) at site 3. Conventions as in Fig 7.

In the most open of our three environments (Site 3, Fig 1B) where large trees are located 10 to 20 m away from the reference location the transIDFs are shallower compared to Site 1 and Site 2 (Fig 8). Except for an image difference increase in NW corner of our sampling grid, the IDFs are comparatively symmetrical in different directions but become very shallow at different heights (Fig 8B and 8C).

### The catchment volumes of panoramic snapshots

The range over which a given reference snapshot can provide navigational guidance back to the location at which it was taken depends on the sensitivity with which a navigating agent, such as a homing insect can detect how the image difference between the memorised snapshot and the current view changes as the agent moves. One way of determining this range for any given habitat and insect of interest is thus to assess the local gradient of image differences throughout the panoramic image cube we constructed and through this procedure map the locations where that gradient exceeds a threshold value. This threshold gradient only allows us to describe the information potentially available to a navigating agent employing a hill climb or gradient descent in image differences. At this stage, we do not know the sensitivity with which insects, or other animals, can determine image differences.

**Fig 9:**
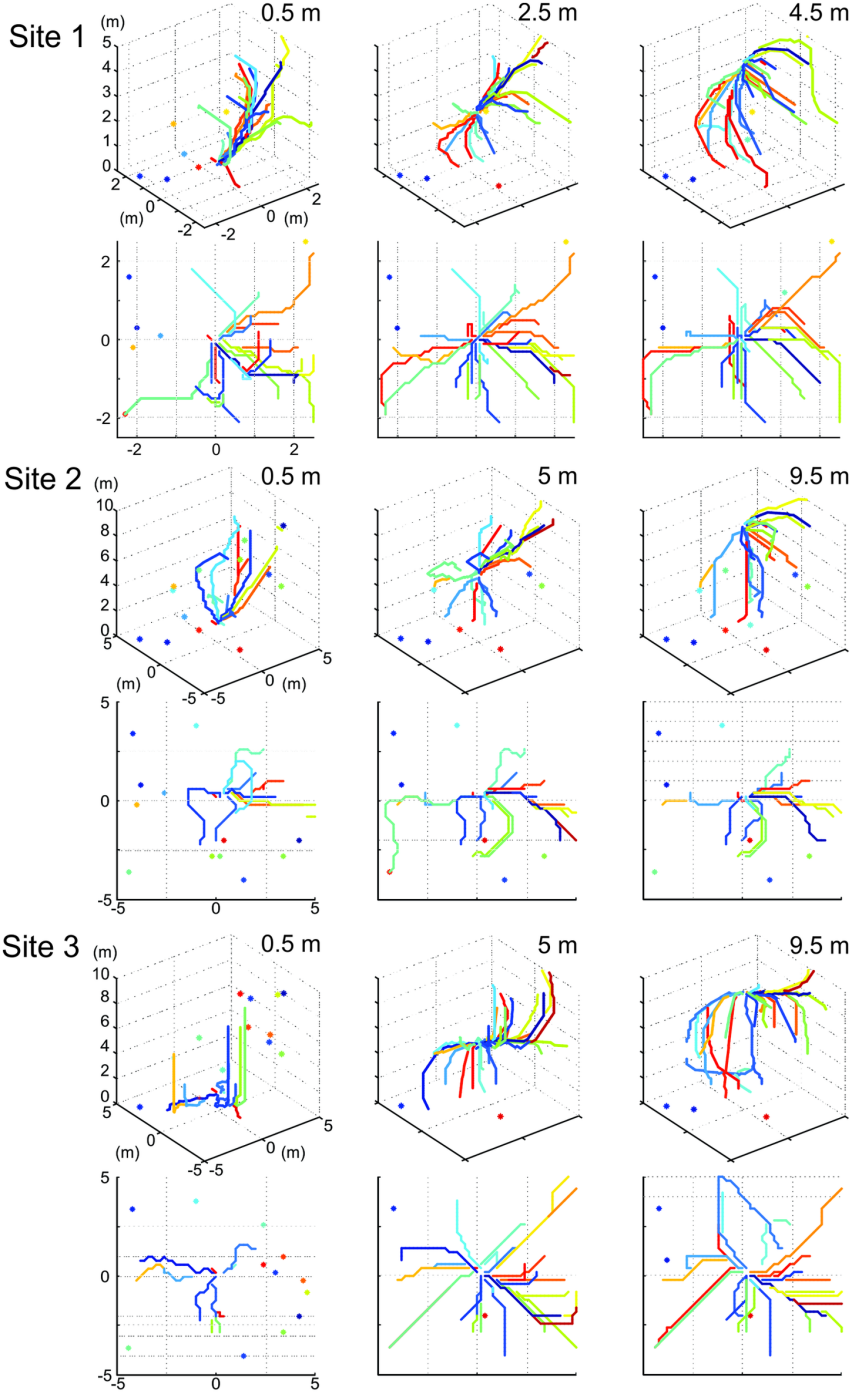
The paths of a hill climbing algorithm at three sites for three reference image heights. Panels show the paths generated by a hill climbing algorithm from 30 randomly chosen locations within the panoramic image cubes at our three sites. Lines pass through spatial locations of each grid-point of a path that successfully reaches the reference image location. Grid-points that are not reached by hill climbing are marked by asterisks. Top rows for each site show the isometric view looking NE. The bottom rows show the top-down views of paths.

To confirm that this procedure indeed identifies continuous gradients in space, we ran both hill climbs and gradient descents (albeit without a threshold) from randomly chosen locations within the panoramic image cubes (see Fig 9). The paths these two algorithms produce and the catchment volumes that result from their systematic application are consistent and comparable (see S1 Fig).

**Fig 10:**
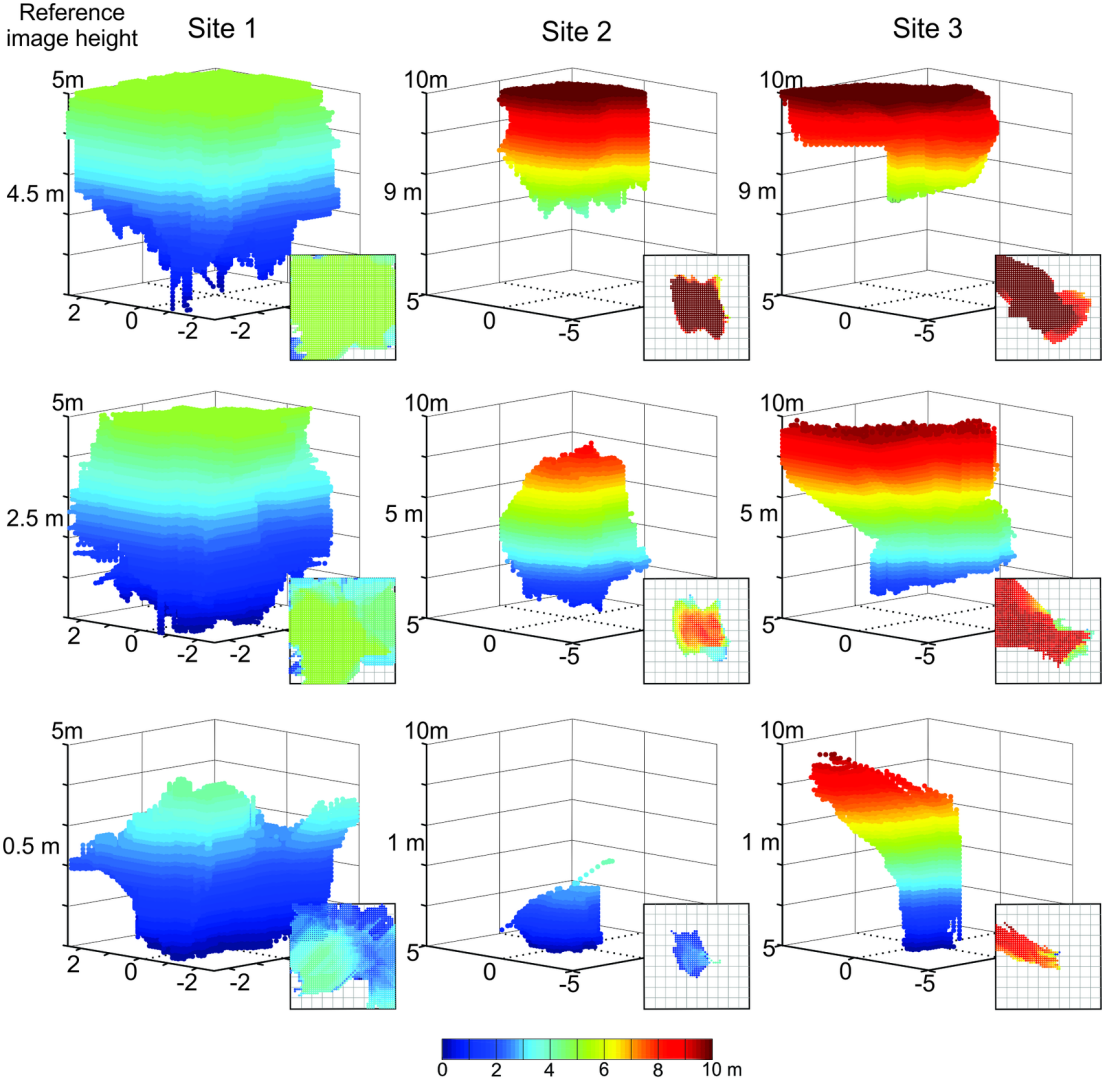
The catchment volumes of panoramic snapshots. Panels show the volumes within which a hill climb of image differences would successfully lead into the reference image location for reference images at different heights above ground from close to ground-level (bottom row) to several metres above ground (top row) as indicated by numbers on the left for the three Sites (left to right column). Catchment volumes contain a voxel (3D pixel) for each grid point for which we found a local IDF slope of more than 10% of the maximum rotational image difference of the reference image per meter. In addition, the voxel needed to be part of a path leading to the reference image location (see Methods for details). Volumes are false-colour coded according to height above ground (see colour bar). Insets show top-down view of each volume.

We plot the catchment volumes we determined by hill climbing with threshold for reference snapshots at three different heights above ground in our three reconstructed habitats in Fig 10. The catchment volume is a 3D structure that contains a voxel (3D pixel) for each grid point from which we found a continuous gradient into the reference location by ensuring the image difference between neighbouring grid points (in any direction) exceeded the threshold at each step. We used 10% of the rotational image difference of the reference image as a gradient threshold per meter of translation (e.g. Site 1: 0 m = 2.90/m, 2.5 m = 3.66/m. 5 m = 3.43/m, where the maximum possible difference would be 255 for 8 Bit grey level images). These volumes thus define the space over which an agent could find its way back to the reference location using purely translational movements provided it can determine image differences with a sensitivity of 10% of the rotational image difference of the memorised image per meter of translation.

The panels in Fig 10 show the catchment volumes around reference images taken 0.5 m, 2.5 m (5 m) and 4.5 m (9.5 m) height above ground (bottom to top row, Fig 10) at our three sites (left to right column). Note that the catchment volumes for Site 1 could only be determined within a 5 m^3^ cube, due to the closeness of vegetation.

Comparing the catchment volumes at our three sites clearly shows that firstly, these volumes are large, extending over several metres and secondly that their volume and shape are determined by habitat structure, in particular the 3D layout of landmarks. The latter property causes volumes to become larger as the distance to the ground of reference images increases and to vertically elongated shapes in the case of Site 3, where distant and large trees dominate the habitat. The reason for these elongated volumes is an elongated tree-shadow line on the ground.

### The effects of ground-features and of the distant panorama

The views we rendered in 3D models of natural environments are only approximations to the views experienced by navigating animals. We explore in the following two potential sources of artefacts associated with rendered model views: one is the effect of strong shadows on the ground and is inevitably associated with the particular time of day at which image series are recorded, and the second is the limited spatial extent of 3D models (see Methods) which leads to the exclusion of visual features in the very distant panorama.

**Fig 11:**
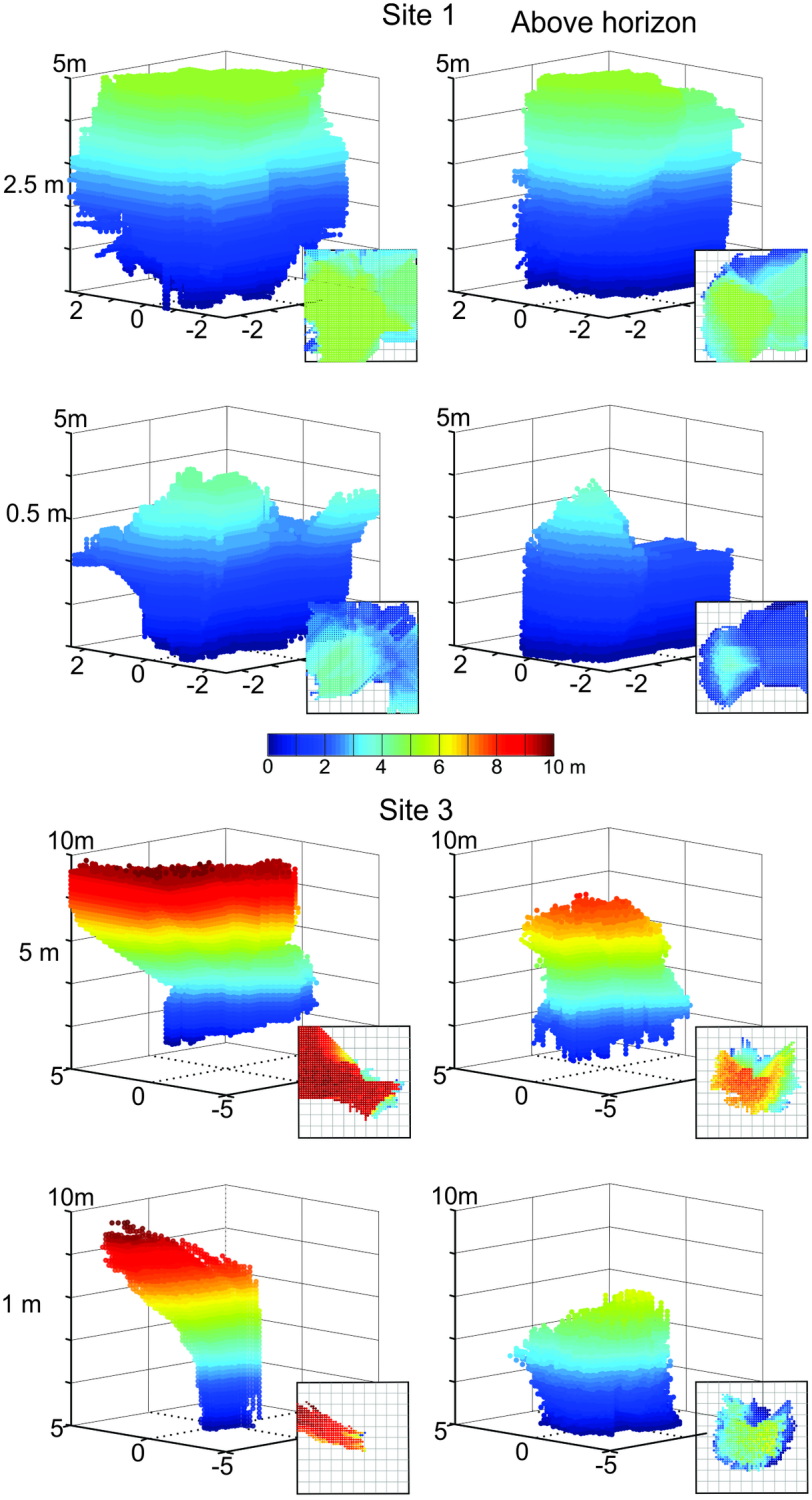
The effect of ground features on catchment volumes. Panels show catchment volumes based on panoramic images with (left column) and without the parts of the scene below the horizon (right column) at Site 1 (top panels) and Site 3 (bottom panels) for different reference image heights as indicated by numbers on the left. Otherwise conventions as in Fig 9.

**Fig 12:**
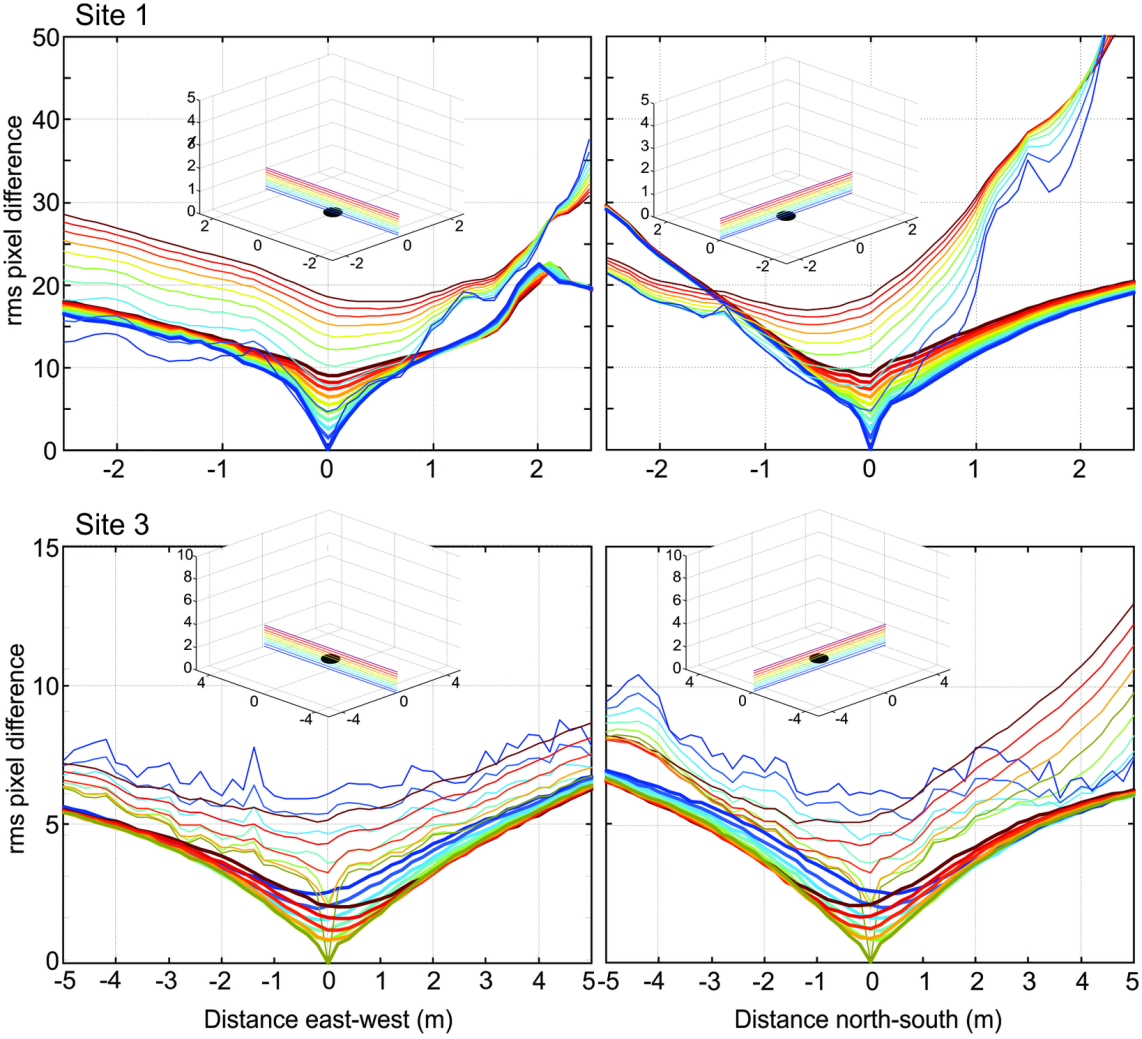
The effect of ground features on the image difference functions (transIDF) close to the ground at site 1 and site 3. For the reference image at ground level, east-west and north-south transects through the transIDF are shown at 10 or 20 cm height intervals as indicated by insets. The transects with the ground plane removed are shown in bold and overlayed over the transects with the whole image used at the same site and height.

To examine the degree to which features on the ground affect translational image differences we generated new catchment volumes by only comparing the parts of scenes that are seen above the horizon (Fig 11). The way in which catchment volumes are affected by the removal of close features on the ground is surprising and counter-intuitive: in most cases (shown for two reference image heights at Site 1 and Site 3 in Fig 11) catchment volumes become smaller when only above-horizon features are considered and their shape changes radically (e.g. 1 m reference image height at Site 3, Fig 11). A detailed consideration of transIDF transects close to the ground (Fig 12) suggests that removing features on the ground from comparison images makes gradients smoother by removing false minima, but at the same time also making them flatter (due to only distant objects contributing to the scene, see [2]. This flattening is most notable in the vertical direction especially at the edges of the volume. These shallow gradients lead to hill climbing paths that have gentler slopes resulting in catchment volumes that are smaller, because many local gradients failed to exceed the 10% rotIDF threshold we have set (S2 Fig).

**Fig 13:**
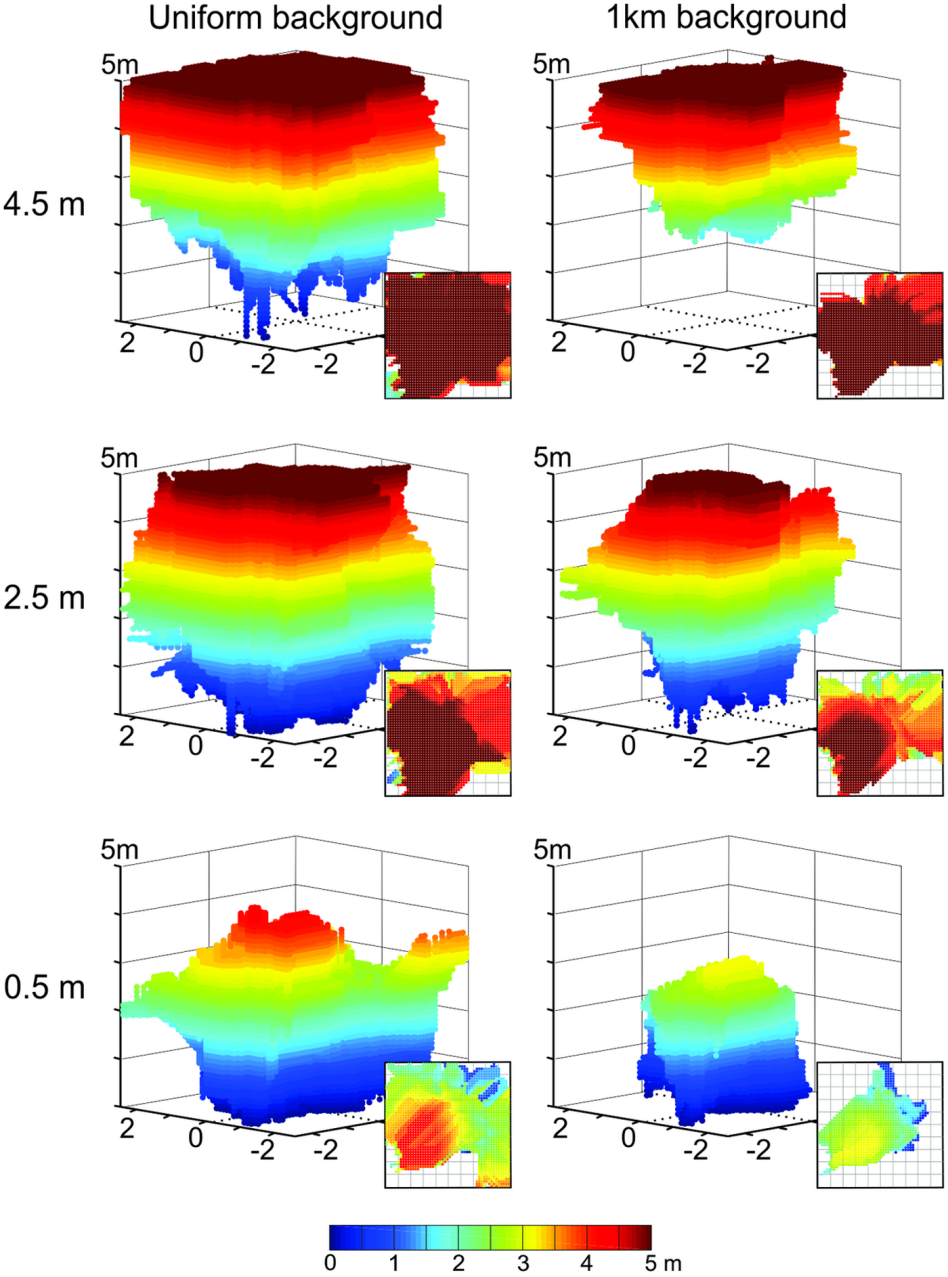
The effect of distant panorama features on catchment volumes. Panels show catchment volumes based on panoramic images with (right column) and without a photographic panoramic background at 1 km distance (left column) for three different reference image heights (top to bottom rows) at Site 1. Otherwise conventions as in Fig 10.

We tested the effect of distant panorama features by projecting the panorama as seen from the centre of Site 1 onto a sphere at a distance of 1 km (see Methods). Comparing catchment volumes with and without such a distant panorama (Fig 13), shows again surprisingly that distant background features lead to a constriction of catchment volumes. This reduction in extent appears to be due to the darker backdrop, which leads to smaller r.m.s pixel differences and a shallower transIDF than the brighter blue used in previous treatments.

## Discussion

We have described the catchment volumes within which panoramic images provide navigational information to the location of a reference image in natural scenes. We define navigational information as the presence of a continuous gradient in image differences between current views and a reference image. Catchment volumes in natural environments are surprisingly large, stretching over several metres, depending on the 3D layout of landmarks in the environment and thus are habitat-specific. We add the caveat that the practical size and shape of these catchment volumes depend on the sensitivity of navigating agents to panoramic image differences and also on the way in which images are pre-processed by early vision computations (e.g. [2]) or represented in the brain (e.g. [10, 19, 23]).

In what follows we discuss first the limits of current methods to map navigational information in natural environments and secondly, suggest in what way our findings are relevant to studies of spatial orientation and navigation.

Initially panoramic imagers were used on board a robotic gantry to investigate how image differences develop with distance from a reference location [1]. This method is slow and with the equipment available at the time was restricted to one cubic metre of natural space. An obvious way of extending the range of this method and to make it more versatile would be to use panoramic imagers on board small quadrocopters, as they become increasingly available. However, a number of problems would need to be solved: the imaging device would need to be stabilized against roll and pitch, the flying platform would need to be flown along accurately controlled paths using Differential GPS and image capture would need to be synchronized with accurate location information, although it may be possible to recover camera locations from image series alone, provided the panoramic imagers in use can be calibrated. In addition, images very close to the ground are difficult to capture with this method.

The alternative method at present is to construct 3D models of natural environments, either with laser-scanner-colour-camera combinations, or with purely camera-based methods, such as the one we used here and to render panoramic images within these models [3]. This approach has the advantage of allowing a systematic exploration of space, including locations very close to the ground, but suffers from a number of other problems, such as the limited horizontal and vertical range of such models (e.g. laser scanner ca 80m; camera-based methods even less due to limitations of horizontal and vertical camera movements required for triangulation) and reconstruction artefacts due to environmental motion generated by wind-driven plant movements and time-of-day dependent shadows on the ground.

All these methods suffer from three more general problems: at present we lack the ability to fully quantify navigational information along the dimensions of the spectral sensitivities of animals, with the exception of the utility of UV/Green contrast for navigation [24–26]. In addition, the dynamic range of visual systems is much larger than the 8bit images we currently use for reconstruction and lastly, our current methods cannot sufficiently capture the dynamics of natural scenes due to changes in illumination, the movements of clouds and shadows, although there is no principal reason why they cannot be adapted to systematically investigate these dynamics.

### Catchment volumes and visual navigation

The surprisingly large range over which panoramic snapshots can provide navigational guidance, not only for walking animals (e.g. [3, 14]) but also in three dimensions, as we have documented here, go some way to explaining the abilities of honeybees to navigate over 100s of metres (reviewed in [27]). Successful navigation of honeybees at this scale depends on the bees having performed orientation flights in the same landscape [28, 29] during which they are likely to memorize changing views as ants do along novel routes (e.g. [30]) and bees, ants and wasps do during their learning flights and learning walks close to the nest (e.g. [31–33]). We have shown here that gradients become smoother as reference images are collected further away from the ground and that catchment volumes become larger, potentially allowing insects to recognize and approach a location from several meters away. It would be interesting to know the height at which bees fly during their orientation flights, because it would allow us to reconstruct catchment volumes and predict the range over which acquired views provide navigational information (e.g. [29]). The same opportunity exists for exploring the visual information available to navigating birds that fly at heights between 10s and 100s of metres, where we would predict catchment volumes to be very large (see, for instance [34]). The reason being that the transIDF catchment becomes larger with increasing distance of the closest objects (see [2]) and the average distance of the closest visual features on the ground scales with height above ground. In addition, with increasing height above ground and less occlusion, prominent features of the distant panorama become visible for 10s to 100s of kilometres.

Given that locally and closer to the ground (in the range between 0.5 m and 9.5 m which we investigated here) catchment volumes extend for metres, it is interesting to note that nesting insects spend so much time during their learning flights and walks looking into the nest direction from many different compass directions (e.g. [31, 35, 36]). Why is a single snapshot not enough? One possibility is that insects need to be able to find the nest from all compass directions, because they cannot predict where they would find food, water or nest materials. It may also be the case that remembering views at different heights above ground, at different distances and bearings from the nest and with the nest in defined positions in the visual field makes homing more efficient. Rather than relying on gradient descent in image differences, homing insects may simply associate familiar views which they had seen across the nest from different compass directions during learning walks and learning flights with the nest direction as has been suggested for ants and ground-nesting wasps [31, 36]. This may reflect the fact that very close to the ground, translational image difference functions are very noisy, as we have shown, or to say it differently, the useful gradient into the reference location only extends for a few centimetres, beyond which the image difference ‘landscape’ has many false minima. The sources of this visual aliasing near the ground in natural environments require more detailed investigation, because it is not immediately obvious that there would be particularly repetitive features such as rocks and grass patches that could cause this ‘noise’.

We have also shown that the ‘noise’ from false minima and maxima near the ground plane can be eliminated by only comparing the part of the scene above the horizon or by acquiring views at different heights above ground. Removing the ground from panoramic scenes appears to have several effects: it eliminates the noise in the transIDF near the ground plane by smoothing irregular changes in image differences and by removing the steepest gradients, which in every case we investigated were due to shadows on the ground. However, gradients also become shallower overall (as shown in Fig 12), which leads to more symmetrical, compact, and smaller catchment volumes at least when assuming that an agent has a gradient detection threshold.

There are a number of ways in which insects could cope with ‘shadow noise’: they may attend to above horizon features only until very close to the goal by using the panorama sky contrast (e.g. [8, 9, 24–26]) and/or could employ local contrast normalization that practically removes the effects of shadows and changes in illumination [2]. However, in pinpointing their nests in the ground, flying insects such as wasps clearly attend to small visual features on the ground (e.g. [31]) and at this stage it is not clear whether this is due to small objects becoming more salient as they are approached, or whether homing insects switch attention from above-ground to on-ground features as they approach the nest.

Finally, the counter-intuitive result requires an explanation that the distant background panorama decreases the size of the catchment volumes, compared to the–rather artificial–situation in which this background is lacking. These distant features on the one hand extend the range over which the rotational image difference function has a detectable minimum, but at the same time, image differences due to translations are now generated not against a homogeneous bright background, but against darker visual features. The result is a shallower translational difference gradient and a smaller catchment volume, compared to a situation where distant background features are absent. We add the caveat that these effects depend on the detection threshold used, which in turn is likely to depend on pixel noise or environmental noise levels such as wind-driven vegetation that limit detection of image differences due to rotation and translation.

### Outlook

The catchment volumes of panoramic views in natural scenes describe a fundamental property of the world by defining the range over which such views provide navigational information about location. Much remains to be investigated about how these volumes depend on habitat structure, on the sensitivity of animals to image differences, on different kinds of noise levels and on the different ways of parsing, segmenting and representing natural scenes that animals may employ. For instance, there is growing evidence that both flying and walking insects use the skyline to determine their bearing (e.g. [8, 9, 37, 38]) and we have shown here how this affects catchment volumes by removing the effects of ground shadows and ground feature noise. However, for accurate localization insects do need to attend to features that provide guidance in the centimetre range and there is a need to test at what distance from reference locations insects are guided by ground feature details (e.g. [31]). This would clearly also be of interest for researchers developing visual guidance algorithms for autonomously navigating robots. Lastly, the reconstruction and analysis tools we introduced here would need to be applied to build larger models of more diverse habitats to investigate the relationship between habitat structure, catchment volumes and optimal strategies for location and route learning.

## Supporting information

S1 Supplementary Information Supporting methods for gradient descent and ground plane removal. (DOCX)

S1 Fig A comparison between hill climbing and gradient descent algorithms. Shown are the catchment volumes (top rows) and example paths (bottom rows) for three reference image heights at Site 2 (columns) as determined by hill climbing (HC, top) and gradient descent algorithm (GD, bottom). Paths start at 30 randomly chosen grid-points within the panoramic image cube. We visualised paths by drawing a line through the spatial location of each grid-point (for HC), or each step location (GD) of a path that successfully reached the reference image. We defined success for gradient descent as coming to within 99% of a grid-point to the reference image. Grid-points from which an algorithm does not reach the reference image location are shown as asterisks. Catchment volumes show all grid-points from which the algorithm reaches the reference image location.
(TIF)

S2 Fig The effect of removing the ground plane on the slope along the path of hill climbs. Panels show the paths of failed hill climbs up to the point at which they became sub-threshold. The left panes show failures at Site 3 with the full panorama, and the right panes show the failures for the same site when we removed the pixels below the panorama’s horizon. The top panes show an isometric view, while the bottom panes show the top-down view. Lines show paths that would make it to the reference image if there were no threshold, while asterisks show grid-points that left the volume or converged on false local minima.
(TIF)
