# Supplementary material for: Quantifying navigational information: The catchment volumes of panoramic snapshots in outdoor scenes
Source: PLoS One. 2017 Oct 31;12(10):e0187226. doi: 10.1371/journal.pone.0187226 (PMC5663442; doi:10.1371/journal.pone.0187226)
Supplement: S1 Supplementary Information — (DOCX) [file pone.0187226.s001.docx]

**Supplemental Information**

**Path finding algorithms**

We used a hill-climbing algorithm to determine the catchment volume of panoramic images since it allowed us to implement a slope threshold representing an agent’s visual acuity. However, since gradient descent algorithms are the standard path finding algorithm used in animal navigation research we have generated and compared catchment volumes using gradient descent to those produced by hill climbing (without a threshold).

Method

Our gradient descent algorithm determines the slope in each of the three spatial dimensions at a given spatial location and moves in the direction of each slope a distance equal to the gradient. The gradient descent catchment volumes show each grid-point from which a gradient descent successfully reached within 99% of a grid-point (0.099m for Site 1, 0.198m for sites 2&3) of the spatial location of the reference panorama before 1000 steps. We compared these two algorithms at all sites and under all treatments (see data store) and show a subset of these results (Fig S1).

We determine the gradient in each dimension at a particular 3D position within the volume by building a 3x3x3 cube of coordinates whose corners represent the smallest grid-point cube that encompasses the position. For each of the 12 edges we calculate the transIDF at a point equivalent the offset of the 3D position along the dimension of that edge. We then calculate the transIDF for each of the six faces taking the offset in the new dimension as an intermediate between two neighbouring edges. Finally, we find the transIDF gradient in each dimension by finding the difference in the transIDF between the two opposing face values along each dimension.

Result

Although there are slight differences between the catchment volume of the hill climbing algorithm and the gradient descent algorithms these differences are minor. Generally, the gradient descent leads to smaller volumes, but the difference is usually between 0-2 grid-points (Figure S1). We think these methods are very comparable and lead to the same conclusions with respect to our findings.

**Figure S1.** **A comparison between hill climbing and gradient descent algorithms.** Shown are the catchment volumes (top rows) and example paths (bottom rows) for three reference image heights at Site 2 (columns) as determined by hill climbing (HC, top) and gradient descent algorithm (GD, bottom). Paths start at 30 randomly chosen grid-points within the panoramic image cube. We visualised paths by drawing a line through the spatial location of each grid-point (for HC), or each step location (GD) of a path that successfully reached the reference image. We defined success for gradient descent as coming to within 99% of a grid-point to the reference image. Grid-points from which an algorithm does not reach the reference image location are shown as asterisks. Catchment volumes show all grid-points from which the algorithm reaches the reference image location.

Discussion

The catchment volumes from gradient descent are slightly smaller than those generated by hill climbing, but they are largely similar. It should be noted that there are some grid-points from which gradient descent is successful whereas hill climbing is not and *vice versa*. Since the results of hill climbing and gradient descent are largely similar when determining the maximum potential catchment volume, we recommend using gradient descent when determining the theoretical maxima but recommend using hill climbing when trying to account for the visual acuity of an agent. Furthermore, for cases where the transIDF is generated in a discrete grid we recommend hill climbing, since it is best suited for discrete data as in our case, while gradient descent is best suited when continuous data are available.

**Removing the ground plane**

In order to understand the effect ground features below the horizon of the panorama have on the catchment volume of reference images we compared the slopes of hill climbing paths with and without the below horizon pixels. We find a general decrease in magnitude of the mean slope of hill climbing paths when we removed the ground plane. This decrease in the magnitude of the slope means that more paths are sub-threshold, fail to reach the reference image and so the size of the catchment volume is smaller by comparison. This is the opposite effect to the apparent increase in the size of the catchment volume when no threshold is used (compare the number of asterisks in Fig. S2).

**Figure S2.** **The effect of removing the ground plane on the slope along the path of hill climbs.**  Panels show the paths of failed hill climbs up to the point at which they became sub-threshold. The left panes show failures at Site 3 with the full panorama, and the right panes show the failures for the same site when we removed the pixels below the panorama’s horizon. The top panes show an isometric view, while the bottom panes show the top-down view. Lines show paths that would make it to the reference image if there were no threshold, while asterisks show grid-points that left the volume or converged on false local minima.
