# Supplementary figures and images for: Quantifying navigational information: The catchment volumes of panoramic snapshots in outdoor scenes
Source: PLoS One. 2017 Oct 31;12(10):e0187226. doi: 10.1371/journal.pone.0187226 (PMC5663442; doi:10.1371/journal.pone.0187226)

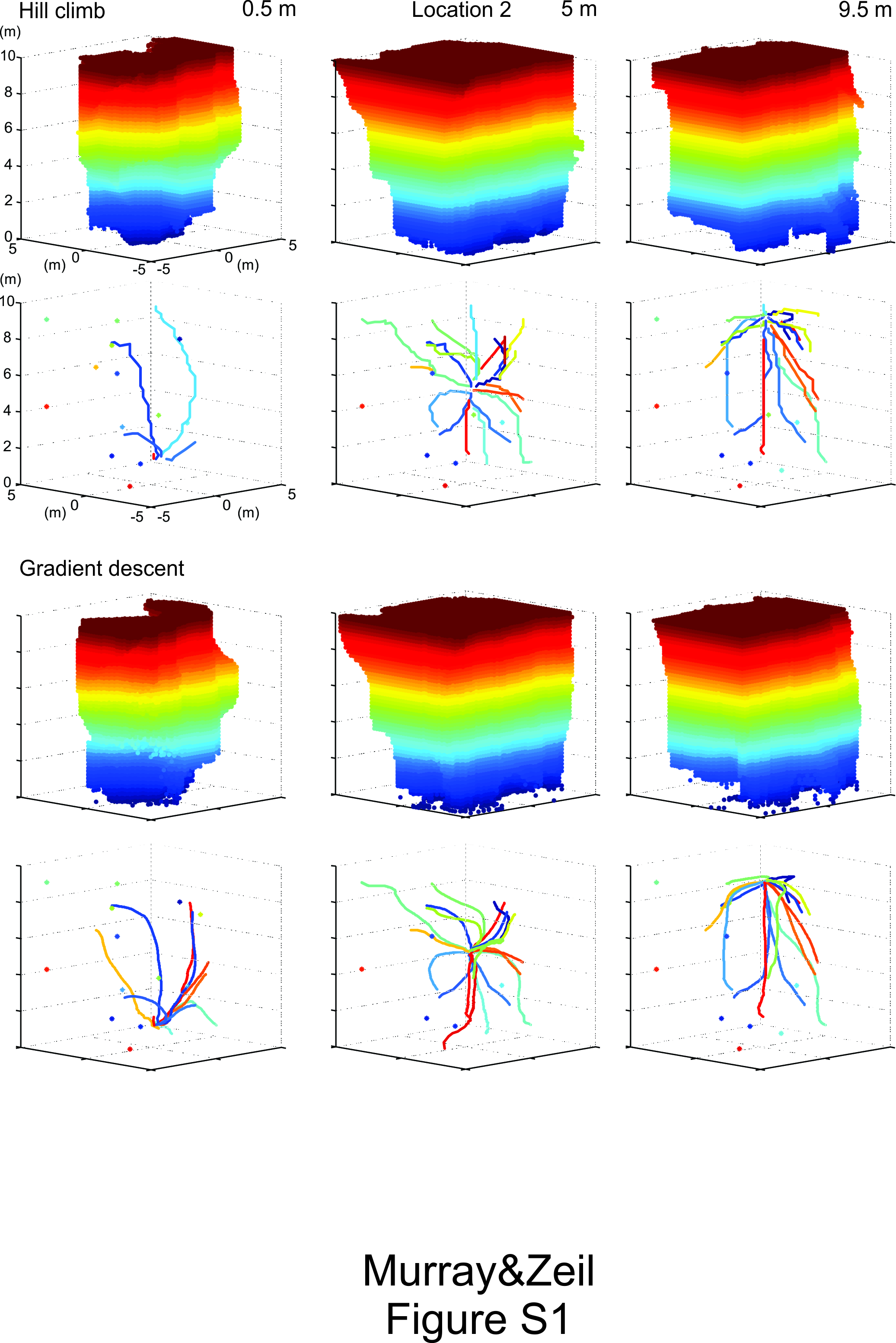

Supplement: S1 Fig — Shown are the catchment volumes (top rows) and example paths (bottom rows) for three reference image heights at Site 2 (columns) as determined by hill climbing (HC, top) and gradient descent algorithm (GD, bottom). Paths start at 30 randomly chosen grid-points within the panoramic image cube. We visualised paths by drawing a line through the spatial location of each grid-point (for HC), or each step location (GD) of a path that successfully reached the reference image. We defined success for gradient descent as coming to within 99% of a grid-point to the reference image. Grid-points from which an algorithm does not reach the reference image location are shown as asterisks. Catchment volumes show all grid-points from which the algorithm reaches the reference image location. (TIF) [file pone.0187226.s002.tif]

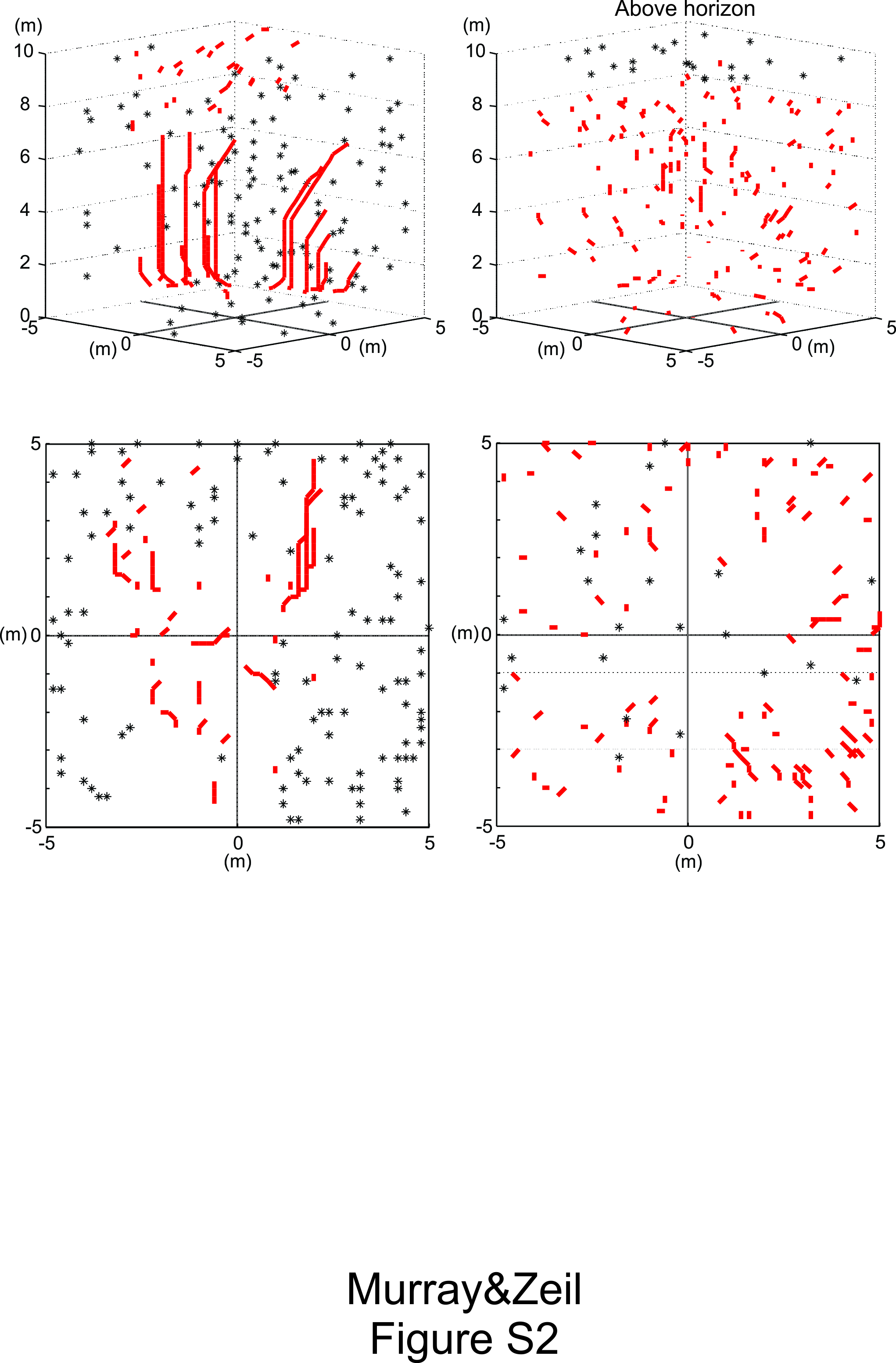

Supplement: S2 Fig — Panels show the paths of failed hill climbs up to the point at which they became sub-threshold. The left panes show failures at Site 3 with the full panorama, and the right panes show the failures for the same site when we removed the pixels below the panorama’s horizon. The top panes show an isometric view, while the bottom panes show the top-down view. Lines show paths that would make it to the reference image if there were no threshold, while asterisks show grid-points that left the volume or converged on false local minima. (TIF) [file pone.0187226.s003.tif]
